# Increased Basal Ganglia Modulatory Effective Connectivity Observed in Resting-State fMRI in Individuals With Parkinson’s Disease

**DOI:** 10.3389/fnagi.2022.719089

**Published:** 2022-03-08

**Authors:** Nicholas J. Wapstra, Micah Ketola, Shelby Thompson, Adel Lee, Tara Madhyastha, Thomas J. Grabowski, Andrea Stocco

**Affiliations:** ^1^Department of Radiology, University of Washington, Seattle, WA, United States; ^2^Center for Molecular and Behavioral Neuroscience, Rutgers University, Newark, NJ, United States; ^3^Department of Kinesiology, University of Georgia, Athens, GA, United States; ^4^Etosha Business and Research Consulting, Mount Berry, GA, United States; ^5^Amazon Web Services, Seattle, WA, United States; ^6^Department of Neurology, University of Washington, Seattle, WA, United States; ^7^Department of Psychology, University of Washington, Seattle, WA, United States

**Keywords:** common model of cognition, resting-state fMRI, Parkinson’s disease, dynamic causal modeling, basal ganglia

## Abstract

Alterations to interactions between networked brain regions underlie cognitive impairment in many neurodegenerative diseases, providing an important physiological link between brain structure and cognitive function. Previous attempts to characterize the effects of Parkinson’s disease (PD) on network functioning using resting-state functional magnetic resonance imaging (rs-fMRI), however, have yielded inconsistent and contradictory results. Potential problems with prior work arise in the specifics of how the area targeted by the diseases (the basal ganglia) interacts with other brain regions. Specifically, current computational models point to the fact that the basal ganglia contributions should be captured with modulatory (i.e., second-order) rather than direct (i.e., first-order) functional connectivity measures. Following this hypothesis, a principled but manageable large-scale brain architecture, the Common Model of Cognition, was used to identify differences in basal ganglia connectivity in PD by analyzing resting-state fMRI data from 111 participants (70 patients with PD; 41 healthy controls) using Dynamic Causal Modeling (DCM). Specifically, the functional connectivity of the basal ganglia was modeled as two second-level, modulatory connections that control projections from sensory cortices to the prefrontal cortex, and from the hippocampus and medial temporal lobe to the prefrontal cortex. We then examined group differences between patients with PD and healthy controls in estimated modulatory effective connectivity in these connections. The *Modulatory* variant of the Common Model of Cognition outperformed the *Direct* model across all subjects. It was also found that these second-level modulatory connections had higher estimates of effective connectivity in the PD group compared to the control group, and that differences in effective connectivity were observed for all direct connections between the PD and control groups.We make the case that accounting for modulatory effective connectivity better captures the effects of PD on network functioning and influences the interpretation of the directionality of the between-group results. Limitations include that the PD group was scanned on dopaminergic medication, results were derived from a reasonable but small number of individuals and the ratio of PD to healthy control participants was relatively unbalanced. Future research will examine if the observed effect holds for individuals with PD scanned off their typical dopaminergic medications.

## Introduction

Parkinson’s disease (PD) is a neurodegenerative syndrome that targets predominantly dopaminergic neurons in the substantia nigra pars compacta (SNc; Dauer and Przedborski, 2003; Jankovic, 2008). As the SNc provides dopamine to the striatum along the nigrostriatal pathway, a decrease in dopamine results in dysfunctional basal ganglia function. Reduced dopamine input to the basal ganglia has been associated with impaired motor function, specifically in neurodegenerative diseases like PD. Contemporary accounts of basal ganglia function (Frank et al., 2004; Stocco et al., 2010) suggest that they work by controlling, or “gating”, the influx of signals from other cortical areas to the prefrontal cortex. The basal ganglia achieve this *via* two main pathways, commonly referred to as the direct and indirect *anatomic* pathways. The direct *anatomic* pathway has an excitatory effect on the prefrontal cortex and is composed of striatonigral neurons expressing D1 receptors. The indirect *anatomic* pathway has an inhibitory effect on the prefrontal cortex and is composed of striatonigral neurons expressing D2 receptors. Both D1 and D2 receptors are modulated by dopamine but have opposite responses in line with the pathways in which they reside; dopamine has an excitatory effect on D1 receptors, and an inhibitory effect on D2 receptors. In PD, depletion of dopamine to the striatum translates into an upregulation of the indirect *anatomic* pathway and downregulation of the direct *anatomic* pathway, resulting in more conservative gating of cortical signals to the prefrontal cortex (Albin et al., 1989). In terms of the brake–accelerator model, there is a tendency to favor the brake over the accelerator (Albin et al., 1989; DeLong, 1990). Further understanding of basal ganglia function in PD is vital to interpret the mechanism of the disorder and its effect on functional connectivity in the brain.

Although it is most intuitive to think about measuring network function during tasks (when cognitive networks are specifically recruited), most brain activity occurs spontaneously and in the absence of specific stimuli and reflects activity in task networks. Functional magnetic resonance imaging (fMRI) measures brain activity by detecting changes associated with blood flow which is coupled with neuronal activation. Blood flow to a brain region increases when that region is in use. Resting-state fMRI (rs-fMRI) is integral in the identification and investigation of networks within the brain. rs-fMRI consists of continuous recordings of brain activity while participants are awake, but not engaged in any task. Analysis of rs-fMRI data has shown that intrinsic activity has a rich spatiotemporal structure, reflecting how networks of cortical regions combine and recombine over time. These networks inform us about the innate rhythms and oscillations in healthy and disordered brains, and abnormality in the dynamics of these networks has been reliably associated with neurodegenerative diseases, psychiatric disorders, and aging (Sambataro et al., 2010; Hohenfeld et al., 2018).

Despite the previous success of rs-fMRI in related disease and aging fields, attempts to characterize the effects of PD on network functioning using rs-fMRI have yielded inconsistent and contradictory results. While somestudies did identify functional connectivity abnormalities related to the basal ganglia (Hacker et al., 2012; Szewczyk-Krolikowski et al., 2014), other studies failed to do so (Amboni et al., 2015; Gao and Wu, 2016) even if the disease originates precisely in this circuit. We hypothesized that a potential problem with prior work is the difficulty of capturing the specifics of second- order interactions. Initial choices made about how functional connectivity is analyzed have important consequences for their ultimate outcome. For example, the most common approach in functional connectivity analysis relies on measuring pairwise correlations between pairs of regions; the degree to which changes in metabolic activity in one region occur in synchrony with changes in activity in another region is taken as a proxy of their functional relatedness (Göttlich et al., 2013; Baggio et al., 2015). If, however, the function of the basal ganglia is to gate signals between cortical regions, then pairwise correlations are unlikely to discover any abnormalities. Rather, abnormal basal ganglia function would manifest itself in second-order effects, with changes in basal ganglia activity associated with changes in the correlations between regions. Therefore, measures of cortico-cortical connectivity are biased by underlying patterns of basal ganglia activity. This has been empirically verified in patients with PD (Hammond et al., 2007; Schroll and Hamker, 2013; Lebedev et al., 2014).

These second-order modulatory effects require a different methodological approach. One such method is Dynamic Causal Modeling (DCM; Friston et al., 2003). DCM estimates effective (that is, directional) connectivity between pairs of regions by iteratively refining the parameter estimates of a dynamic system, in which brain regions are approximated as neural mass points. DCM is a generative technique: it proceeds from an initial network based on a selected theoretical model of cognitive function, and iteratively refines its connectivity parameters until they are best matched with the observed time courses. The relationships between the network nodes can include both first order (direct) and secondorder (modulatory) connections, thus making this method uniquely adapt to the case of PD.

The power afforded by DCM, however, comes at a price. In contrast to most functional connectivity analyses, DCM is a purely “top-down”, model-based technique, and requires an initial theoretical model of network connectivity that is fit to the data.

**Figure 1:**
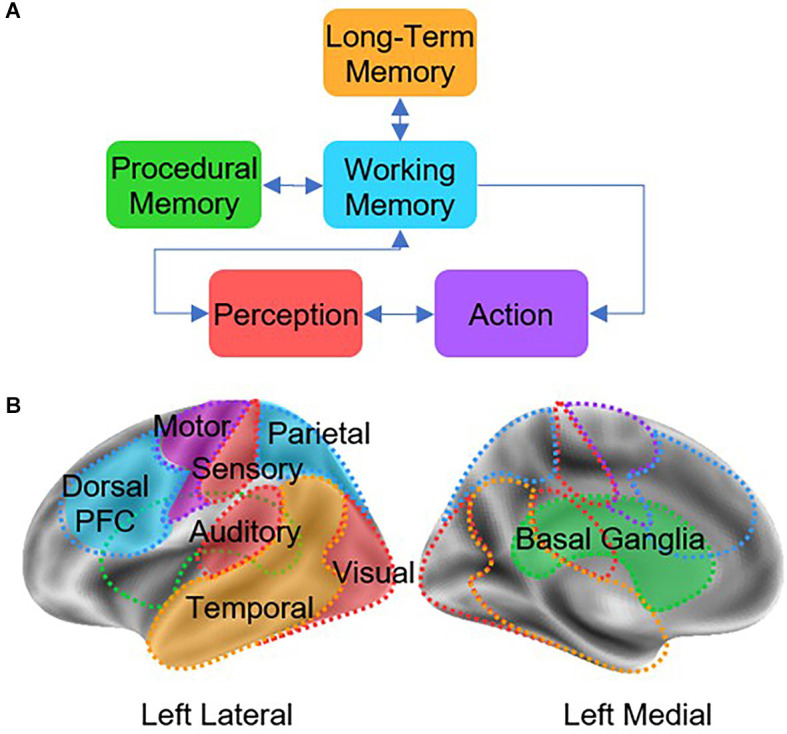
**(A)** Architecture of the Common Model of Cognition (CMC), as described by Laird et al. (2017). **(B)** Theoretical mapping between CMC components and homologous cortical and subcortical regions.

Because final estimates of connectivity depend on the larger network model in which a connection is embedded, we adopted a minimal but reasonable and biologically plausible network architecture that captures the large-scale interactions between brain regions. The selected model is named the Common Model of Cognition (CMC; Laird et al., 2017), and representsa consensus architecture for general intelligence that serves as a blueprint to understand the organization of a human-like mind (Laird et al., 2017). There are five functional components in the CMC that can be mapped to anatomical regions: long-term memory (hippocampus and medial temporal lobe, MTL), working memory (prefrontal cortex, PFC), procedural memory (basal ganglia, BG), perception systems (visual, auditory, and sensory cortices, SENS), and action systems (motor cortex, MC; see Figure 1A). Although the CMC is a purely *functional* architecture, whose components are characterized in terms of abstract computations, researchers have mapped the components of the CMC onto homologous brain regions (see Figure 1B) and translated the relationships between CMC components onto predicted patterns of functional connectivity (Steine-Hanson et al., 2018; Stocco et al., 2018). An investigation usingmultiple neuroimaging recordings from 200 individuals in the Human Connectome Project (Sibert et al., 2021) has shown that, when used as a network model for connectivity analysis, the CMC provides a better fit to the data than any of 12 other large-scale architectures (six based on a hierarchical organization and six based on a “hub-and-spoke” network design) derived from the literature. Furthermore, the CMC’s provided a superior across six different tasks spanning different domains (working memory, relational reasoning, decision-making, emotional processing, language, social cognition), a fact that confirms its wide applicability as a large-scale, systems-level neural architecture.Thus, by estimating the effective connectivity of the basal ganglia within the larger network of the CMC, we are maximizing the rigor of the analysis and the generalizability of our conclusions.

Importantly, the CMC provides theory-driven hypotheses about the functional relationship between brain regions with clear connections to PD. The loss of dopamine neurons in PD has cognitive consequences that can be computationally characterized (Frank et al., 2004) and successfully modeled in cognitive architectures (Stocco, 2018).

**Figure 2:**
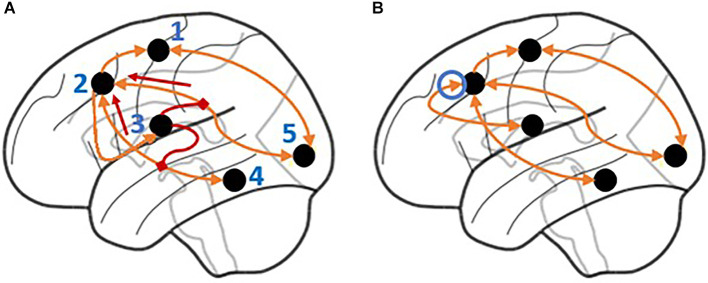
Illustration of the signal flow for the *Modulatory* and *Direct* Model. **(A)** In the *Modulatory* Model, the basal ganglia modulate the signals flowing through incoming connections to the prefrontal cortex. The connection between the prefrontal cortex and basal ganglia is unidirectional with no direct connection from the basal ganglia to the prefrontal cortex. Short arrows highlight the directionality of the modulatory connections. **(B)** In the *Direct* Model, the basal ganglia have a direct (and bidirectional) connection to the prefrontal cortex. The circled arrow highlights the connection that is only present in the *Direct* model. Regions are labeled as follows: 1. Motor cortex, 2. Prefrontal cortex, 3. Basal ganglia, 4. Hippocampus and medial temporal lobe, 5. Visual, auditory, and sensory cortices.

Two variations of the CMC have been put forward that reflect two theories of basal ganglia function. The first is the *Direct* CMC (not to be confused with the direct *anatomic* pathway), in which all regions of interest (ROIs) are directly connected, and effective connectivity is measured across these direct connections (see Figure 2B). The second is the *Modulatory* CMC, which incorporates additional assumptions that capture our modern understanding of the basal ganglia, the brain region associated with the CMC’s procedural memory component. This model replaces the direct connection between the BG to the PFC with two second-level, modulatory connections that control projections from the MTL to the PFC and from the SENS to the PFC (see Figure 2A). Although both implementations are in principle compatible with the tenets of the CMC (Laird et al., 2017), the use of modulatory connections reflects a contemporary functional interpretation of the role of the basal ganglia; according to this view, the basal ganglia do not directly manipulate the contents of working memory, but rather “gate” (Frank et al., 2004) or “route” (Stocco et al., 2010) information from other areas to the prefrontal cortex. It is important to note that, in this context, the term “modulatory” only refers to the non-linear, multiplicative effect of the basal ganglia on cortico-cortical connectivity. This effect captures the complex interplay of cortico-cortical and thalamo-cortical synaptic activity within a mathematical formalism, and should not be confused with the neuromodulatory effect of dopamine: although dopamine is depleted in PD, its neuromodulatory effect in the basal ganglia is not supposed to be altered by the disease, and is not captured at the level of analysis of functional connectivity. A recent re-analysis of the data of the HCP study that showed the superiority of the CMC (Sibert et al., 2021) provided preliminary evidence that, at least in healthy young adults, the modulatory version of the CMC provides a better fit than its direct, non-modulatory counterpart.

Based on the existing literature, two hypotheses were examined:

Our first hypothesis is that the DCM analysis will confirm that the *Modulatory* CMC will provide a better fit to the fMRI data than the *Direct* CMC, across all participants. This hypothesis is suggested by the fact that the modulatory model better captures the functional role of the basal ganglia, as seen in contemporary computational models (Frank et al., 2004; O’Reilly and Frank, 2006).

Our second hypothesis is that, within the *Modulatory* model, a difference will be found between PD patients and controls in the parameters that describe modulatory connectivity. Specifically, we predict that modulatory signals will be *weaker* in PD patients than in controls. This hypothesis follows from our knowledge that PD restricts the gating of cortical signals to the prefrontal cortex (Albin et al., 1989), which, in DCM, would be reflected in a *lower* or *negative* value of the BG modulatory connections.

## Materials and Methods

### Participants

Participants were recruited from a larger, multimodal study of functional networks in typical PD. As part of a comprehensive protocol involving EEG and MRI, 113 participants received a resting fMRI scan. Participants were monitored using eye-tracking for wakefulness. Of these, two participants were excluded based on a diagnosis of dementia (PD = 2). Among the 111 remaining participants (PD: *N* = 70, Age = 67.92 ± 8.12, Female = 26; Controls: *N* = 41, Age = 69.87 ± 8.92, Female = 16), 106 were also enrolled in the Pacific Udall Center (PUC) Clinical Core. The PUC PD group was ascertained by referral from local neurologists and the Washington State Parkinson Disease Registry (WPDR; Kim et al., 2018). All participants from the PUC PD group who were included in this study met UK Parkinson’s Disease Society Brain Bank clinical diagnostic criteria for PD (Goetz et al., 2007). All members of the PUC control group had no signs of Parkinsonism on examination. No such data were available from the five participants (PD = 4, non-PD = 1) who did not take part in the PUC Clinical Core and their diagnosis of PD was provided by a movement disorder specialist (PD = 1) or by community neurologists (PD = 3). All patients with PD were judged to have early or mid-stage idiopathic PD, as evidenced by a Hoehn & Yahr stage of 3 or less. The control group included spouses of PD patients, community volunteers, and participants designed as “controls” in the WPDR. All PUC participants underwent: (1) a neurological examination and the Movement Disorder Society-sponsored revision of the Unified Parkinson’s Disease Rating Scale (MDS-UPDRS) PartIII (Gibb and Lees, 1988) performed by a movement disorder neurologist, (2) detailed cognitive testing includes the Montreal Cognitive Assessment (MoCA), and (3) a structured interview to collect data on demographics, medication use, and clinical history.

Data collected from PUC participants were discussed at a diagnostic consensus conference to assign a cognitive diagnosis (normal or dementia) using procedures previously described (Cholerton et al., 2013). Two participants with PD were diagnosed at a consensus with dementia. PD and control groups did not differ significantly in cognitive testing using the MoCA (PD: MoCA = 26.18 ± 2.27, Controls: MoCA = 26.30 ± 2.89, *p* = 0.83).

Participants with PD completed the fMRI measurements while on their prescribed PD medications. Of the 67 participants with PD, 15 were taking levodopa without a dopamine agonist, two were taking dopamine agonists without levodopa, 44 were taking both levodopa and a dopamine agonist, one was taking levodopa, a dopamine agonist, and a cholinesterase inhibitor, and five were taking neither levodopa nor a dopamine agonist. Levodopa equivalent daily dose (LEDD) was collected for all participants.

This study was approved by the University of Washington Institutional Review Board and all participants provided written informed consent.

### Image Acquisition and Processing

MRI data were acquired on a research-dedicated 3T Philips Achieva whole-body scanner (Philips Medical Systems, R5.1.7) with a 32-channel SENSE head coil at the Integrated Brain Imaging Center of the University of Washington, Seattle. Functional data were acquired while participants were instructed to lay quietly and focus on a fixation cross, using a gradient echo-planar multi-echo pulse sequence with TR = 2, 500 ms, a 79° flip angle, and TE = 9.5/27.5/45.5 ms. Multi-echo recordings allow for increased sensitivity and a reduced number of artifacts (Power et al., 2018). Each volume acquisition consisted of 37 oblique axial slices, each of which was 3.5 mm thick with 0-mm gap and contained 64 × 64 voxels with an in-plane resolution of 3.5 × 3.5 mm.

Two sets of functional neuroimages were acquired, one continuous run of resting-state data and one of the task-based data. During the task-based data, participants performed multiple trials of a visual discrimination task. The task-based data was only used to identify group-level seed coordinates for the main nodes in the connectivity network examined in the resting state data, and will not be analyzed here (Barch et al., 2013).

During the resting state data, participants were asked to keep their eyes open while a fixation cross was presented on the screen, and were not required to engage in any specific cognitive activity.

In addition to functional images, a T1-weighted structural scan was acquired as an anatomical reference (1-mm isotropic multiecho MP-RAGE: Sagittal TR = 10.019 ms, TE = 4.61 ms, FoV = 260 × 260 × 189.6 mm, and an 8° flip angle).

Resting-state fMRI data were processed using a combination of FSL, AFNI, and SPM. Functional data underwent slice-timing correction, motion correction and realignment, removal of baseline drift, and then spike detection and removal. From there the data were co-registered to the skull-stripped T1-weighted structural scan, normalized to the MNI ICBM152 stereotactical space, and smoothed using a 3D Gaussian filter with 8-mm full-width half maximum.

### Bilateral Regions of Interest

**Figure 3:**
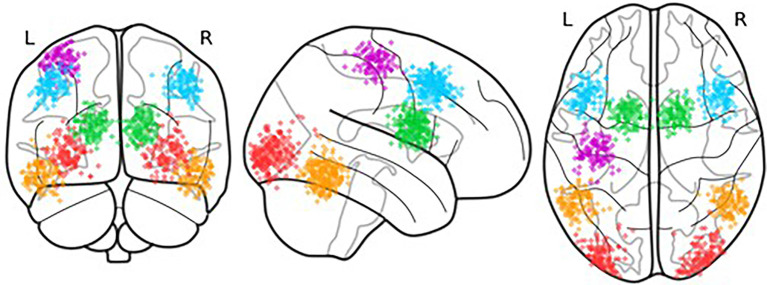
Location of the centroids of each individual ROI. Each point represents the centroid of one ROI; variations in the centroids account for individual differences in functional anatomy.

The ROIs were selected by translating CMC components into anatomical regions. To characterize each component, the ROI coordinates were selected based on the functional data of an attention-based task from the same participants that were collected as part of the larger study, thus accounting for individual differences in functional neuroanatomy (Stocco et al., 2021). This procedure proceeds by first identifying the candidate regions in a group-level analysis of all data. To account for individual differences in functional neuroanatomy, the coordinates of each ROI were localized on an individual brain by identifying the peak of functional activity that is closest to the centroid of the group-level ROIs. The distribution of the centroids of the individual-level ROIs is visually represented in Figure 3. Departing from our previous work that has focused on left-hemisphere regions (Friston et al., 2003), this study used bilateral, instead of unilateral ROIs. Bilateral ROIs were obtained by combining homologous regions in the left and right hemispheres. By averaging hemispheric regions, we can better capture full-brain effects in PD. As part of the attention-based task, almost all participants used their right hand to respond to the task stimuli and left-hand activity was not present. Therefore, all ROIs were bilateral except for the Action ROI which was lateralized to the left hemisphere.

#### Dynamic Causal Modeling Definition

DCM is composed of both a *neural* model, that receives experimental stimuli and predicts the underlying dynamics of brain activity, and an *observational* model, that takes in the predicted underlying dynamics and outputs predictions of observed brain activity. In the case of fMRI, the neural model is given regions of interest (ROIs) and represents the time course of activity in each region *i* as a nonlinear state equation:


$$\dot{y}=Ay+Cx+\sum_{i}y_{i}\;D\;(i)y$$

**Equation 1**. Nonlinear state equation of the neural model using DCM.

In this equation, *A* defines intrinsic connectivity between different regions (fixed connectivity), *C* defines effects by task inputs, *D* defines the modulatory effects that regions have on the connections between other regions, *x* defines task inputs, and *y* defines brain activity.

The observational model is composed of a hemodynamic model that uses neural activity to cause changes in blood flow, which in turn causes changes in blood volume and the amount of deoxyhemoglobin. From there, the volume of blood and deoxyhemoglobin concentration are entered into an output nonlinearity and give rise to an observed BOLD response (Friston et al., 2003).

#### Dynamic Causal Modeling Comparison

DCM was the preferred methodological approach to apply the CMC over other approaches such as Structural Equation Modeling (SEM) and Granger Causality Modeling (GCM) for three reasons. First, it can account for the temporal dynamics of an fMRI time-series, whereas SEM cannot (Friston, 2011). This is important as without temporal dynamics the data is effectively reduced by one dimension, leaving out a significant source of variability. Second, it allows us to better model directed causal influences, which are implied in the directed arrows between CMC components (Figure 1A). Although GCM *can* disentangle the direction of influence, DCM has proven superior at dealing with the variable nature of the BOLD response timing (Friston, 2009). Finally, DCM, but neither SEM nor GCM, is capable of modeling second-order interactions between nodes in a network, i.e., cases in which a region modulates the connectivity between two other regions. This specific case, as it will be shown, is of particular interest in PD, as it plays a significant role in capturing the nature of basal ganglia function.

#### First-Level Model Setup

The application of DCM to rs-fMRI posed a significant challenge because during rs-fMRI there is no task to be performed nor significant external events driving brain activity. Without any task conditions or an external input to initiate network dynamics, and therefore a null *C* matrix, the DCM would simply remain uninitialized with all parameters left at the default values.

Friston et al. (2014) circumnavigated the problem by creating a deterministic DCM. Their version of resting-state DCM estimates effective connectivity based on second-order statistics rather than on the time-series of activation. This transforms analysis from the computationally expensive issue of estimating hidden neuronal states to the more efficient problem of estimating the spectral density of activity changes (Friston et al., 2014). While more computationally efficient, by using second-order statistics their method can no longer capture temporal dynamics in the estimation of effective connectivity. For this reason, we adopted an alternative procedure proposed by Di and Biswal (2014).

**Figure 4:**
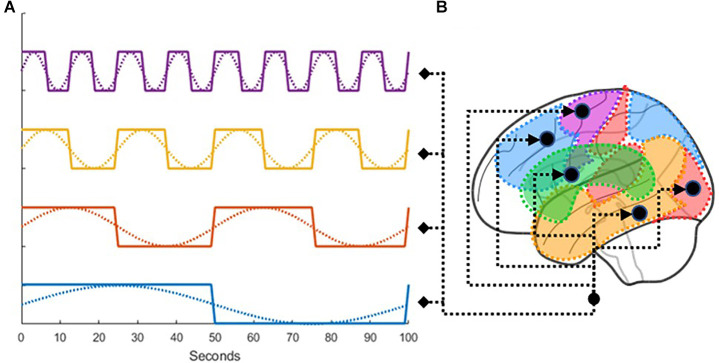
**(A)** Illustration of how low-frequency fluctuations were transformed into boxcar functions, which were then **(B)** used as drivers for the activity in all ROIs.

One of the characteristics of resting-state brain activity is the presence of spontaneous correlations at very low-frequencies, which organize brain networks along different rhythms (Fox et al., 2005). Stephan et al. (2009) explicitly modeled these low-frequency fluctuations (LFF) within the resting-state signal using deterministic inputs. Because this study similarly dealt with rs-fMRI, we borrowed the procedure developed by Di and Biswal (2014), making use of a total of eight boxcar regressors derived from sine waves of different frequencies and phases (Figure 4). We used periodic sine and cosine functions at 0.01, 0.02, 0.04, and 0.08 Hz as task conditions for the *C* matrix. As drivers for activity in DCM cannot be partial, the periodic functions were transformed into boxcar functions. There were two boxcar functions for each frequency with a 90 degree lag in between, at cycles of 100, 50, 25, and 12.5 s. The boxcars were used as input to every node in the analysis replacing traditional task-based inputs.

### CMC Implementations for PD

As the correct interpretation of the role of the basal ganglia within the CMC is crucial to understanding PD, two different interpretations of the CMC were explored and tested across the full set of participants.

In the first model, the *Direct* CMC, all ROIs that are directly connected (see Figure 1A) are implemented as patterns of direct connectivity (i.e., matrix *A*). The remaining model parameters in A in which there are no direct connections are set to 0. Matrix D is set equal to 0 when fitting the DCM for this model because no modulatory effects are examined. Critically, the BG directly projects to the PFC (Figure 2B), reflecting the assumption that procedural knowledge directly manipulates the contents of working memory.

The second model, the *Modulatory* CMC, incorporates additional assumptions that capture our modern understanding of the basal ganglia, the brain region associated with the CMC’s procedural memory component. As its name implies, this model replaces the direct connection between the BG to the PFC with two second-level, modulatory connections that control projections from the MTL to the PFC (Connection 1), and from the SENS to the PFC (Connection 2; Figure 2A). These connections imply the basal ganglia’s modulatory involvement from the temporal lobe and the sensory, auditory, and visual brain regions to the parietal and dorsal prefrontal cortex. Dysfunction in the modulation of these connections could be explanatory for symptoms in the PD group. Thus, in this model, the connection between Procedural and Working Memory in A is set to 0, and the modulatory connections representing Connection 1 and Connection 2 in D are estimated. The difference in the number of parameters between the two models can be set to *n = 1*, which corresponds to the two modulatory parameters in the matrix *D* in Equation 1 minus the direct parameter removed from matrix *A* in Equation 1.

### Statistical Analysis

#### GLM Analysis

We first conducted a general linear model (GLM) analysis to ensure that our oscillatory regressors successfully captured brain activity. To do so, we calculated an omnibus ANOVA across all oscillatory regressors at the participant level. This test captures any variance that can be accounted for by any of the oscillatory regressors. The resulting F-statistic map was then log-transformed, yielding a measure of the difference between the variance explained by regressors and the residual variance (i.e., noise). Finally, a group-level T-test was performed across all subjects on the individual-specific log-transformed F-maps. The result of this analysis is a statistical test of whether the variance captured by the regressors was significantly greater than the variance of the residuals.

#### Bayesian Model Selection

We compare the *Direct* and *Modulatory* models by using Bayesian Model Selection (BMS; Stephan et al., 2009). BMS is implemented by estimating the log group Bayes factor across subjects. The Bayes factor represents the ratio of the likelihoods of two models. The likelihood L of a model m given data y is defined as the probability that m would generate y, i.e., L(m | y) = P(y | m). The log Bayes factor log Bmod, dir represents the log ratio of the likelihood of the modulatory model L(mmod | y) given data y over the likelihood of the direct model L(mdir | y) given the same data. Because of the properties of logarithms, the log of the ration can be simply expressed as log L ( mmod | y ) − log L ( mdir | y). The group likelihood of a model is the joint probability that the model would generate the data of each of n individuals in the group, which can be calculated as Π iP (yi | m) = Π iL(m | yi). Because log Π iL (m | yi) = Σ i log L (m | yi) the log group Bayes factor log Bmod, dir can ultimately be rewritten as:


$$\log\;B_{mid,dir}=\sum_{i\;=\;1}^{n}\log\;L\;(m_{\mathrm{mod}}|y_{i})-\sum_{i\;=\;1}^{n}\log\;L\;(m_{dir}|y_{i})$$

**Equation 2.** Log Bayes Factor equation

where *n* is the number of subjects, *m_mod_* and *m_dir_* represent the modulatory and direct model, respectively (Stephan et al., 2007).

The value of log B_mod,dir_ can be interpreted as thestrength of evidence in favor of one of the two comparative models. In this model comparison setup, the *Direct* model represents the null hypothesis, and the *Modulatory* model represents the alternative hypothesis. A log Bayes Factor of >3 provides moderate evidence for rejecting the null hypothesis and >10 is representative of strong evidence for rejecting the null hypothesis in favor of the *Modulatory* model. A value of <3 represents a failure to reject the null hypothesis and the *Direct* model would be preferred (Lee and Wagenmakers, 2013). *Direct* and *Modulatory* models were compared across all subjects (Dima et al., 2009).

#### Bayesian Parameter Averaging

Bayesian Parameter Averaging (Hoeting et al., 1999; BPA) was used to calculate a Bayesian estimate of direct and modulatory connectivity parameters for each group, respectively. BPA (as implemented using SPM12 software) calculates the posterior mean and variance of each of the modulatory and direct connectivity parameters. These parameters are assumed to be normally distributed. To determine whether modulatory parameter means were lower in the PD group compared to the control group, we tested the following hypothesis:


$$H_{0}:M_{PD}-M_{HC}=0$$

$$H_{A}:M_{PD}-M_{HC}<0$$

where *M*_PD_ is the group distribution of the PD group’s connectivity parameters and M_HC_ is the group distribution of the healthy control group’s connectivity parameters. As an exploratory analysis, we tested the same hypothesis for the *Direct* model connectivity parameters.

## Results

### Regressor Quality Analysis

**Figure 5:**
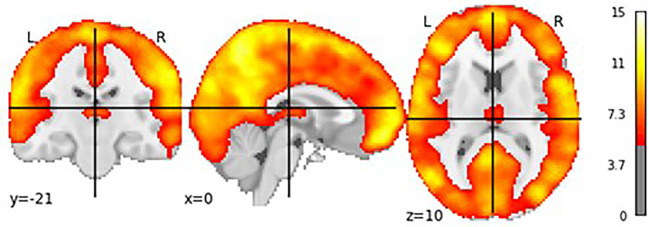
T-test showing voxels whose brain activity was significantly captured by the oscillatory regressors.

Figure 5 shows the results of the group-level *t*-test performed for each participant’s specific log-transformed *F*-map, thresholded at a value of ***t***_(110)_ > 3.166, which corresponds to *p* < 0.05 when corrected for multiple comparisons through the family wise error correction procedure. This test was designed to show how much variance was captured by our eight oscillatory regressors across all voxels (Di and Biswal, 2014). As Figure 5 shows, most of the gray matter voxels exhibited such oscillatory activity. Importantly, the significant voxels in Figure 5 include most of the regions in our predefined ROIs, with the only exception being the subcortical basal ganglia (Figure 1B, visible in the axial and sagittal sections of Figure 4). The lack of effect could be explained by the lower signal-to-noise ratios that are observed in subcortical brain regions in high-density neuroimaging protocols as well as the peculiar BOLD activity of the basal ganglia, which typically spikes in response to reward-signaling events rather than showing sustained spontaneous activity (Delgado, 2007). Note that, since each region’s activity in DCM is affected by the oscillatory regressors as well as the activity of other regions (see Equation 1), the lack of effect in the basal ganglia does not compromise the validity of our DCM analysis as long as the rest of the ROIs show modulation by the oscillatory regressors.

### Model Comparison

To test the first experimental prediction, the log group Bayes factor (see Equation 2) was calculated to compare the *Direct* and *Modulatory* models over all subjects. The value of the log Bayes factor for the model comparison was 100, which, according to Kass and Raftery (1995) represents strong evidence to reject the null hypothesis. This means that, across all participants, the *Modulatory* model provided a better fit to the data and was therefore highly preferred over the *Direct* model.

#### Modulatory Parameter Comparison

Having established the superiority of the *Modulatory* model, we analyzed whether the PD group modulatory connection strengths were weaker for the control group.

**Figure 6:**
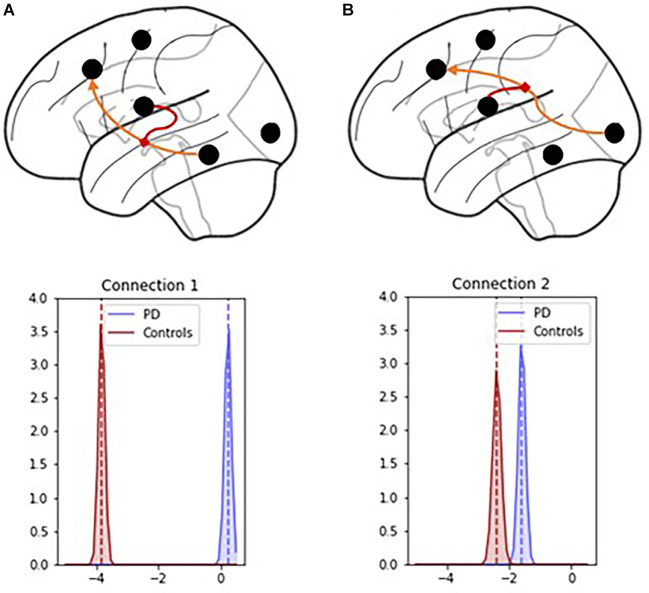
Connection 1 and 2 parameters and their associated posterior probability distributions. **(A)** In Connection 1, the basal ganglia modulate the signals flowing from the hippocampus and temporal lobe to the prefrontal cortex. **(B)** In Connection 2, the basal ganglia modulate the signals flowing from the visual, auditory, and sensory cortices to the prefrontal cortex.

PD modulatory connectivity was found to be positive, or excitatory, and PD patients exhibited higher modulatory effects than controls across Connection 1, which is the modulatory connection of the BG over the MTL to the PFC (see Figure 6 and Table 1). The modulatory connectivity for both the PD and control groups was negative, or inhibitory, and the PD group exhibited higher modulatory effects across Connection 2, which is the modulatory connection of the BG over the SENS to the PFC (see Figure 6 and Table 1).

**Table 1:** Group comparison of modulatory connectivity between PD and Control groups.

Modulatory connection	PD (*n* = 70)		Control (*n* = 41)		M_PD_ − M_HC_ < 0	M_PD_ − M_HC_ > 0
	μ	σ	μ	σ		
MTL to PFC	0.24	0.11	−3.84	0.02	1.00	<0.001
SENS to PFC	−1.59	0.31	−2.39	0.56	1.00	<0.001

*μ = mean; σ = standard deviation, M_PD_ = group distribution PD connectivity parameters, M_HC_ = group distribution of control connectivity parameters, MTL = hippocampus and medial temporal lobe, PFC = prefrontal cortex, SENS = visual, auditory, and sensory cortices*.

The hypothesis test that the PD group had lower modulatory connectivity than the control group (*H*_A_: *M*_PD_ − *M*_HC_ < 0) resulted in p-values of 1 for both modulatory parameters. This indicates that we cannot reject the null hypothesis in this instance.

Based on these findings and the graphical summaries of the modulatory parameters in Figure 6, we examined the hypothesis that the PD group had higher modulatory connectivity than the control group. This hypothesis test results in p-values < 0.001 for both modulatory connections indicating that the null hypothesis can be rejected. The PD group displayed higher modulatory connectivity than the control group across both Connection 1 and Connection 2.

#### Direct Parameter Comparison

In addition to the modulatory connectivity, group differences were also investigated across direct connectivity parameters in the *Modulatory* model, that is, those encapsulated in matrix A of Equation 1. As for the modulatory parameter comparison, we first tested for the hypothesis that the PD group demonstrated weaker effective connectivity than the control group (*H*_0_: *M*_PD_ − *M*_HC_ = 0; *H*_A_: *M*_PD_ − *M*_HC_ < 0). Then, for those parameters for which we could not reject the null hypothesis, we tested the hypothesis that the PD group demonstrated higher connectivity than the control group (*H*_0_: *M*_PD_ − *M*_HC_ = 0; *H*_A_: *M*_PD_ − *M*_HC >_ 0).

The PD group had decreased effective connectivity across SENS to MC, MC to SENS, MTL to PFC, PFC to MTL, SENS to MC, SENS to PFC, PFC to MC, and PFC to BG (*p* < 0.001) compared to the control group. The PFC to SENS connection had a p-value of 1 so we were unable to reject the null hypothesis in this instance. Therefore, we tested the hypothesis that PD had higher connectivity across this connection which was found to be the case (*p* < 0.001, see Table 2).

**Table 2:** Group comparison of direct connectivity between PD and Control groups.

Direct connection	PD (*n* = 70)		Control (*n* = 41)		M_PD_ − M_HC_ < 0	M_PD_ − M_HC_ > 0
	μ	σ	μ	σ		
SENS to MC	−0.16	0.002	0.11	0.006	<0.001	—^1^
MC to SENS	0.07	0.001	0.27	0.004	<0.001	—^1^
MTL to PFC	0.19	0.003	0.31	0.005	<0.001	—^1^
PFC to MTL	0.28	0.003	0.46	0.005	<0.001	—^1^
SENS to PFC	−0.22	0.003	0.10	0.006	<0.001	—^1^
PFC to SENS	0.08	0.002	−0.002	0.004	1.00	<0.001
PFC to MC	0.07	0.002	0.27	0.006	<0.001	—^1^
PFC to BG	0.08	0.004	0.13	0.006	<0.001	—^1^

*μ = mean; σ = standard deviation, M_PD_ = group distribution PD connectivity parameters, M_HC_ = group distribution of Control connectivity parameters, MC = motor cortex, SENS = visual, auditory, and sensory cortices, MTL = hippocampus and medial temporal lobe, PFC = prefrontal cortex, BG = basal ganglia. Note: ^1^ Hypothesis test not completed for this connection*.

## Discussion

Based on the findings, there was clear support for the *Modulatory* model over the *Direct* model which was in-line with our hypothesis. This provides evidence that the *Modulatory* model is better able to capture the functional significance of the basal ganglia.

The direction of the modulatory connection over SENS to the PFC (Connection 2), with PD patients exhibiting inhibition of cortico-cortical connectivity from the basal ganglia, is consistent with the known etiology of the disease (Frank et al., 2004; Stocco et al., 2010). However, the PD group displaying excitatory modulatory connectivity over the MTL to the PFC (Connection 1) and a lower magnitude of connectivity in the SENS to the PFC do not support the prediction of weaker modulatory signals in PD. These findings are unexpected based on the results of previous studies that show the BG, which is impacted in PD, plays a role in information transfer that results in the release of dopamine to modulate workings between SENS to the PFC and MTL to the PFC (Stocco et al., 2010; Schroll and Hamker, 2013). These findings suggest that a compensatory dopaminergic interaction may explain the stronger modulatory connectivity in the PD group. Although we expected the PD group to have decreased connectivity in these connections, taking dopaminergic medications has the effect of reducing the lower range of dopamine values (Stocco et al., 2021). Therefore, the on-medication state of the PD group may explain the counterintuitive findings. Because we expect to see differences in the modulatory connectivity of the BG over PFC connections based on the disruption of the dopaminergic pathway in PD, introducing dopaminergic agents may influence the strength, or even the directionality of our findings. The direct and indirect *anatomic* pathways (see “Introduction” Section) implicated in the gating of signals by the basal ganglia from other cortical regions to the prefrontal cortex would be altered by the introduction of dopamine.

Contrary to the modulatory connections, the PD group had significantly weaker effective connectivity in all direct connections except for PFC to SENS in the *Modulatory* model. This is counter to the direction of group differences observed in modulatory connectivity. While the examination of these parameters was exploratory, the findings suggest that an impairment in the basal ganglia’s function modulation might have downstream effects across multiple cortico-cortical connections. These effects might be partially responsible for the variety of visuo-motor and cognitive problems that emerge in PD as the disease progresses. Furthermore, our results suggest that most of these effects might be invisible in traditional functional connectivity analyses and become apparent only when the modulatory effect of the basal ganglia is properly modeled.

**Figure 7:**
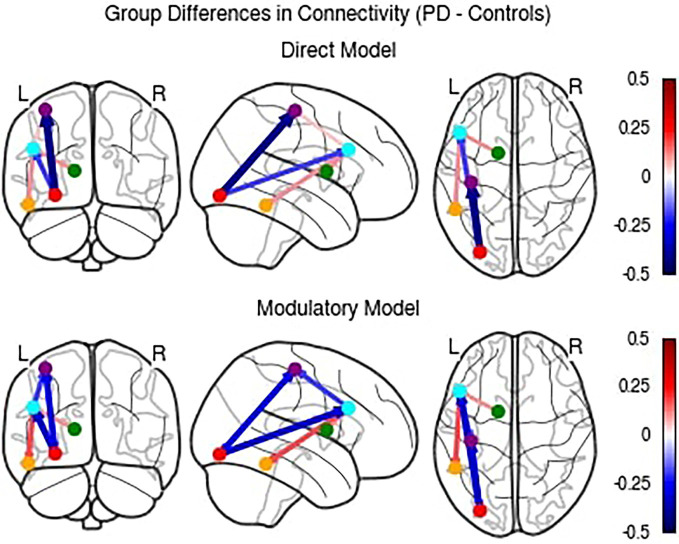
Comparison of differences in direct effective connectivity between PD and Control groups for the *Direct* and *Modulatory* Models.

Because the direct effective connectivity parameters differed from the modulatory connectivity parameters, we completed a *post-hoc* exploratory analysis to examine the difference in direct effectivity parameter values between groups for the *Direct* Model compared to the *Modulatory* Model (see Figure 7).

While the *Direct* Model parameters largely agree with the *Modulatory* Model, there were a few notable differences. The PD group had higher connectivity than the control group in the PFC to MC, PFC to MTL, and BG to PFC connections. These findings indicate that accounting for modulatory connection effective connectivity influences the interpretation of the directionality of the between group results.

Limitations to the findings were that the PD group was scanned on dopaminergic medication. Future research will focus on examining if the observed effect holds for individuals with PD that are scanned off their typical PD medications. Further, results were derived from a reasonable but still small number of individuals (*n* = 111) and the ratio of PD to healthy control participants was relatively unbalanced (70:41). The method used for resting-state DCM also does not allow for dynamic changes in effective connectivity. It uses data from the entire 10-min resting-state scan to create a single account of brain connectivity. We understand that this account is in part naive, as the brain fluctuates throughout resting, and see this as an area for future research.

Limitations notwithstanding, there are clear differences in functional connectivity between PD and controls in resting-state networks. This is demonstrated by the differences across all comparisons of direct and modulatory connectivity. The results also suggest that the *Modulatory* model of the CMC is the correct interpretation of basal ganglia function in which the basal ganglia do not directly manipulate the contents of working memory, but rather route information to other brain regions.

## Data Availability Statement

The raw data supporting the conclusions of this article will be made available by the authors, without undue reservation.

## Ethics Statement

The studies involving human participants were reviewed and approved by University of Washington IRB Committee D. The patients/participants provided their written informed consent to participate in this study.

## Author Contributions

NW: software, validation, formal analysis, investigation, data curation, writing—review and editing, visualization. MK: conceptualization, methodology, software, formal analysis, writing—original draft, and visualization. ST: conceptualization. AL: formal analysis, writing—review and editing. TM and TG: supervision and funding acquisition. AS: conceptualization, methodology, software, validation, formal analysis, writing—review and editing, visualization, supervision, project administration, and funding acquisition. All authors contributed to the article and approved the submitted version.

## Conflict of Interest

AL was employed by the company Etosha Business and Research Consulting. TM was employed by the company Amazon Web Services. The remaining authors declare that the research was conducted in the absence of any commercial or financial relationships that could be construed as a potential conflict of interest.

## Publisher’s Note

All claims expressed in this article are solely those of the authors and do not necessarily represent those of their affiliated organizations, or those of the publisher, the editors and the reviewers. Any product that may be evaluated in this article, or claim that may be made by its manufacturer, is not guaranteed or endorsed by the publisher.
